# Multicomponent bionanocomposites based on clay nanoarchitectures for electrochemical devices

**DOI:** 10.3762/bjnano.10.129

**Published:** 2019-06-25

**Authors:** Giulia Lo Dico, Bernd Wicklein, Lorenzo Lisuzzo, Giuseppe Lazzara, Pilar Aranda, Eduardo Ruiz-Hitzky

**Affiliations:** 1 Instituto de Ciencia de Materiales de Madrid (ICMM), Consejo Superior de Investigaciones Científicas (CSIC), c/Sor Juana Inés de la Cruz 3, 28049 Madrid, Spain; 2 Dipartimento di Fisica e Chimica, Università degli Studi di Palermo, Viale delle Scienze pad 17, 90128 Palermo, Italy

**Keywords:** bionanocomposites, carbon nanostructures, electrochemical devices, halloysite nanotubes, sepiolite

## Abstract

Based on the unique ability of defibrillated sepiolite (SEP) to form stable and homogeneous colloidal dispersions of diverse types of nanoparticles in aqueous media under ultrasonication, multicomponent conductive nanoarchitectured materials integrating halloysite nanotubes (HNTs), graphene nanoplatelets (GNPs) and chitosan (CHI) have been developed. The resulting nanohybrid suspensions could be easily formed into films or foams, where each individual component plays a critical role in the biocomposite: HNTs act as nanocontainers for bioactive species, GNPs provide electrical conductivity (enhanced by doping with MWCNTs) and, the CHI polymer matrix introduces mechanical and membrane properties that are of key significance for the development of electrochemical devices. The resulting characteristics allow for a possible application of these active elements as integrated multicomponent materials for advanced electrochemical devices such as biosensors and enzymatic biofuel cells. This strategy can be regarded as an “a la carte” menu, where the selection of the nanocomponents exhibiting different properties will determine a functional set of predetermined utility with SEP maintaining stable colloidal dispersions of different nanoparticles and polymers in water.

## Introduction

In recent years, the “nanoarchitectonics” concept has helped to develop a large variety of materials with new functionalities [1–6]. Among them, different types of functional materials based on clay minerals have been also prepared; pillared clays and polymer–clay nanocomposites are the best-known examples [7]. Besides classical layered silicates, clays showing other morphologies, such as fibrous (sepiolite and palygorskite) and tubular (halloysite and imogolite) clays, could also be interesting nanoparticulated solids in this context [8–11]. Sepiolite (SEP) and palygorskite are attracting increasing attention in the development of nanoarchitectured materials in applications such as catalysis or biomedicine [8]. The presence of silanol groups at the external surface of the clay fibers allows for the easy assembly with different species facilitating the design and the build up of functional materials. On the other hand, tubular nanoclays, such as halloysite nanotubes (HNTs), are interesting containers for the controlled chemical reactions at nanoscale interfaces and the delivery of active compounds thanks to their unique nature [12], which could be advantageous when integrated as component in nanoarchitectured materials.

Halloysite nanotubes are aluminosilicates of cylindrical shape with the length ranging between 500 and 1000 nm and a lumen diameter between 15 and 70 nm [13]. The lumens represent an ideal nanospace for the uptake and preservation of diverse functional species including drugs, proteins, and enzymes [14–18], even serving as nanoreactor for chemical processes [19]. Of particular interest is the use of HNTs for the uptake of enzymes in an approach for the development of (bio)electrochemical devices like biosensors and enzymatic biofuel cells (EBCs) [20–21]. However, one of the main problems limiting the preparation of HNT-based nanoarchitectured materials is the low colloidal stability of HNTs in aqueous media. This ultimately leads to inhomogeneous and badly performing nanocomposites in spite of the different approaches that have been developed to obtain homogeneous dispersions within different polymeric matrices [22–24]. Therefore, other and more efficient colloidal stabilizers are needed to fully exploit the potential of HNTs.

It has been recently observed that fibrous sepiolite clay mineral of rheological grade (see Experimental section) develops highly stable and viscous suspensions after sonomechanical treatment in water. Dispersions of disaggregated sepiolite can efficiently suspend nanoparticles of different topologies and hydrophobic nature such as graphene nanoplatelets (GNPs) and multiwalled carbon nanotubes (MWCNTs) in water [25–26]. In fact, following this approach it was possible to prepare multifunctional and homogeneous nanocomposite materials such as self-supported sepiolite–nanocarbon hybrid buckypapers [25] and conducting bionanocomposites [26]. Therefore, the present study explores the potential of sepiolite for stabilizing aqueous HNT suspensions.

SEP is a microcrystalline hydrated magnesium silicate with fibrous morphology and dimensions depending on the geological environment of its origin [27]. For instance, an aspect ratio of up to 100 and diameters ranging from 10 to 50 nm are usually observed in sepiolite samples from Taxus basin (Spain) deposits [28]. The unique property of this nanofibrous clay is its ability to largely disaggregate in water after ultrasound treatment, creating thus a rigid, percolated network that can sustain co-dispersed compounds or reinforce polymer matrices [25–26,29]. Interestingly, HNTs are known to maintain their ability to act as nanocontainers even when dispersed in a multicomponent system included in polymer matrices [22]. It has been observed that positively charged polymers such as chitosan (CHI) can electrostatically incorporate the previously loaded halloysite through interactions with its external surface, leaving the lumen unaffected. This offers interesting possibilities for further inclusion of diverse guest species [30–31]. In addition, the role of the polymer matrix is crucial to process advanced bionanocomposite materials either as films or as foams [32–34]. This type of hybrid material offers the advantage of a large interface improving the contact efficiency between the entrapped active molecules and the external environment allowing for the development of promising devices for biosensing [35–36] and enzymatic biofuel cells (EBCs) [37–38].

**Figure 1:**
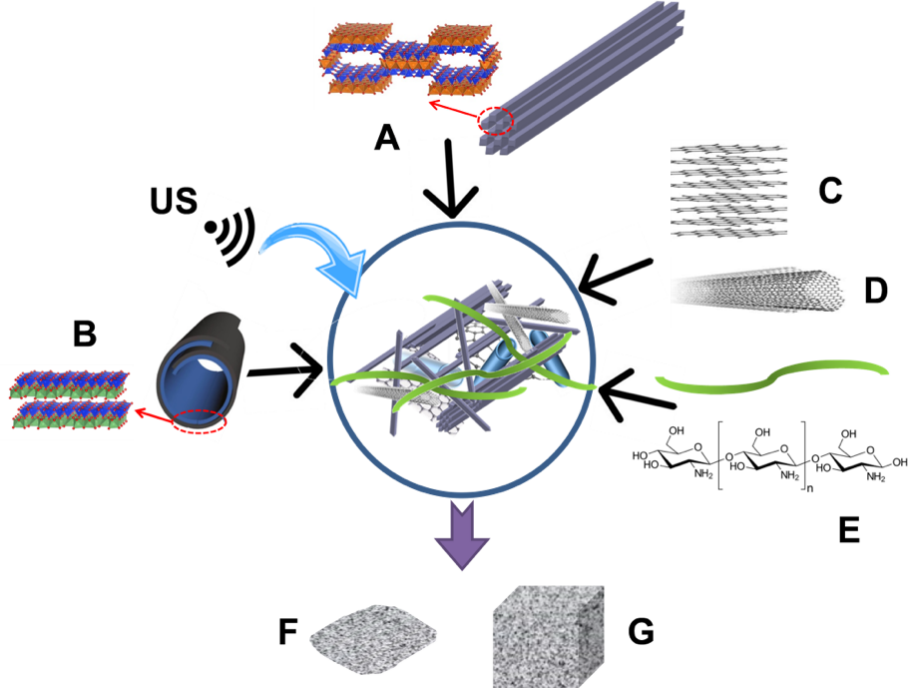
Schematic representation of the different components integrated in the bionanocomposite materials, i.e., (A) sepiolite fibrous clay, (B) halloysite nanotubes, (C) graphene nanoplatelets, (D) multiwalled carbon nanotubes, and (E) chitosan biopolymer, prepared in aqueous media under ultrasound irradiation (US). The resulting nanoarchitectured materials can be formed into films (F) or foams (G).

In this work, conducting multicomponent nanoarchitectured materials involving HNTs, GNPs, MWCNTs, and a CHI matrix were prepared and processed as films and foams from aqueous suspensions of the components dispersed through ultrasound irradiation as schematized in Figure 1. The incorporation of glucose oxidase (GOx) into the lumen of HNTs has been chosen here as an example for the immobilization of bioactive species, which can be crucial to design (bio)electrochemical devices with high performance and long life-time. The SEP, GNP, and MWCNT components are also expected to behave as polymer nanofillers to ensure the mechanical strength and electrical conductivity of the prepared bionanocomposite films and foams [26]. Moreover, MWCNTs are supposed to act as nanowires improving the contact between the active site of the immobilized enzymes and an electrode surface via direct electron transfer [39].

The resulting multicomponent systems have advantages such as high electrical conductivity and flexibility that make the bionanocomposite films appropriate components for biosensors [35,40] for glucose detection, while the relatively high porosity of the bioactive foams enhances the power density and operational stability of EBCs [37,41].

Herein, the performance of the biosensor was evaluated by cyclic voltammetry exploiting the mediated electron transfer (MET) mechanism and the power density of the assembled biofuel cell is examined through polarization curves obtained with linear sweep voltammetry in the direct electron transfer (DET) mode.

## Results and Discussion

### Preparation of bionanocomposite films and foams

**Figure 2:**
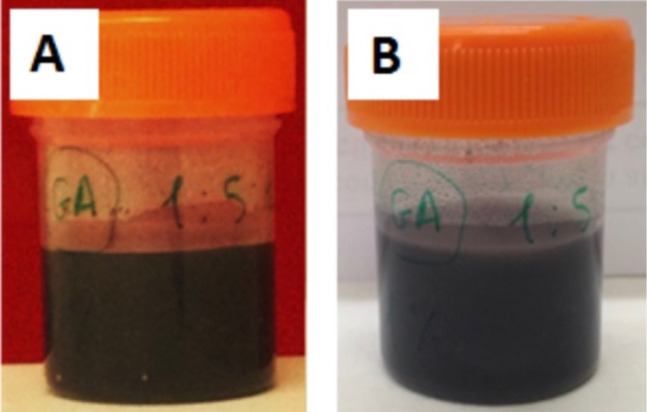
Photographs of dispersions of 1 wt % of a multicomponent bionanocomposite (composition of sample Film-1 given in Table 2); A) freshly prepared and B) after five months.

The preparation of multicomponent nanoarchitectured materials used as functional nanofiller in the further preparation of bioactive and conducting nanocomposites was carried out by mixing of SEP and HNT nanoclays with GNPs and MWCNTs in aqueous media assisted by sonomechanical treatment as schematized in Figure 1. The generation of homogeneous and stable multicomponent dispersions in water (Figure 2) can only be accomplished thanks to the rheological properties of the SEP fibrous clay (Pangel^®^ S9) under ultrasound irradiation.

**Figure 3:**
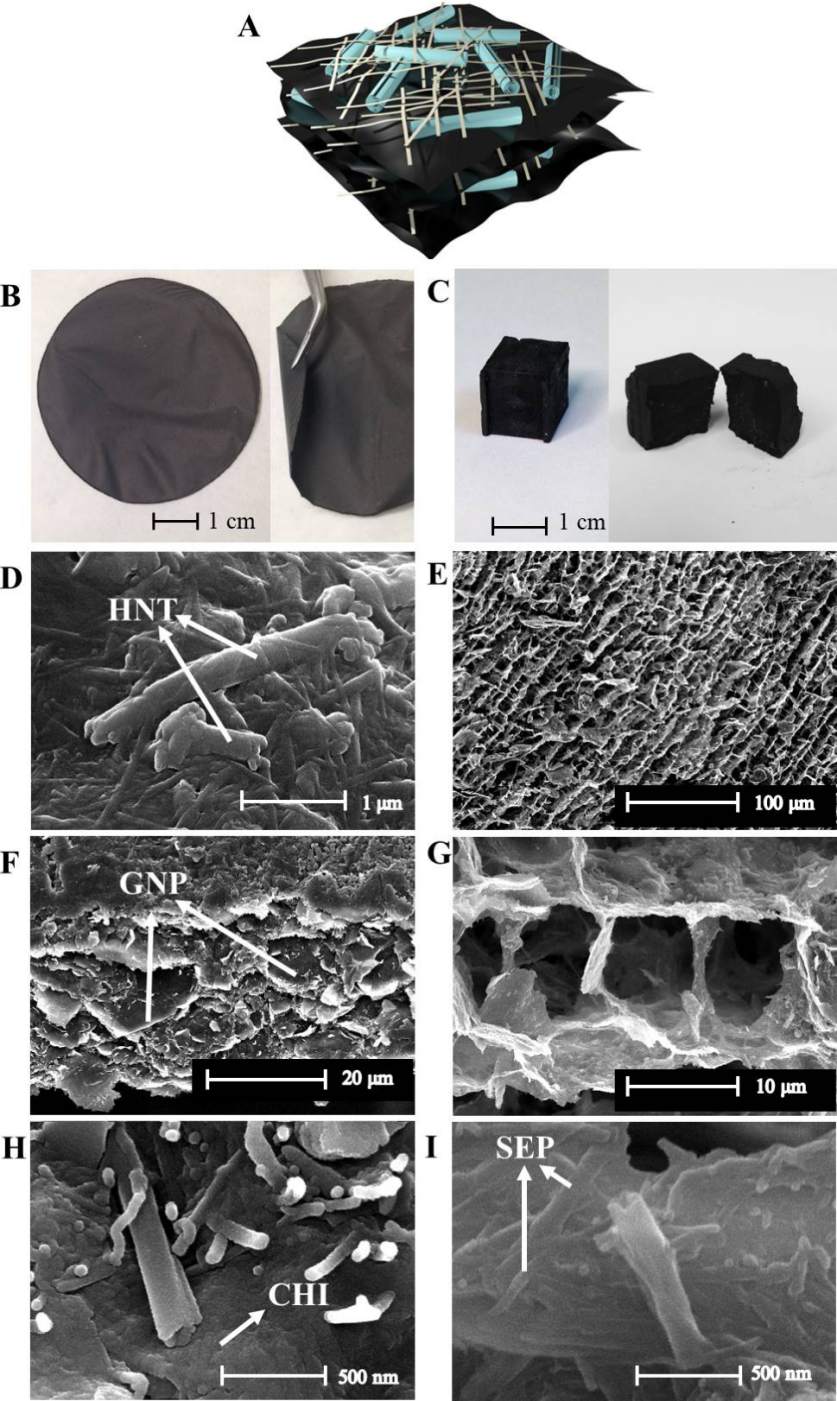
Schematic representation of particle assembly in the multicomponent bionanocomposites: A) cross section of processed materials: HNTs and SEP are represented as tubes and fibres, while chitosan, GNPs and MWCNTs are depicted in the black matrix. Photographs of B) Film-1 and C) Foam-1. SEM micrographs of the film: D) upper surface, F) and H) cross section; SEM micrographs of the foam: E) and G) pore architecture, I) cell walls.

The incorporation of these components into a polymeric CHI matrix results in composite materials that can be processed either as films or as foams. In agreement with previous works [25–26], the ultrasound treatment of this type of sepiolite in aqueous medium promotes the homogeneous dispersion of diverse nanoparticulated components. It can be inferred that the disaggregated fibres of sepiolite form an interpenetrated network representing, in the present case, a steric hindrance for GNPs, MWCNTs, and HNTs to aggregate. This avoids phase segregation and particle sedimentation. These dispersions remained stable for more than five months (Figure 2B) and proved to be suitable for preparing self-supported, flexible films by solvent-casting (Figure 3B) as well as foams by freeze-casting (Figure 3C).

The lamellar arrangement of the bionanocomposite films is schematized in Figure 3A, while SEM images (Figure 3D,F) reveal that the components are uniformly distributed throughout the film and are organized as a compact particle assembly within the chitosan matrix. Furthermore, the film cross section (Figure 3F) displays the typical layered structure of films solvent-cast from fibre dispersions [26,29,42]. Importantly, the access to the lumen of the HNTs appears to remain unblocked despite the assembly with the other components (Figure 3H), which is crucial for the effective use of HNTs as nanocontainers for bioactive molecules. The presence of MWCNTs was not detected in the SEM images given their small size and low concentration (2–5%) in the bionanocomposites.

Freeze-casting rendered foams of high uniformity and shape fidelity (Figure 3C). The foams display open, cell-like pores (Figure 3E,G) with a pore diameter of 13 ± 4 μm and a cell wall thickness of 0.2–0.4 μm (Figure S1, Supporting Information File 1), comparable to similar freeze-cast clay nanocomposite foams [43–44]. Halloysite nanotubes are visible on the surface of the cell walls with free access to the lumen (Figure 3I).

The porosity of the foams was estimated from their relative density values (Table 1). It was found that foams with a high content of chitosan showed the lowest porosity, i.e., 89%. The porosity of films with low chitosan content was 96%. In fact, by reducing the chitosan content (and concomitantly increasing the clay and GNP content) the apparent density slightly decreases, while the skeletal density increases due to the higher density of the solid components. Consequently, the relative density decreases and the porosity increases. It is interesting to note that the foam structure does not seem to collapse after reducing the polymer content, which would otherwise lead to higher apparent density values. The increased apparent density of the foams at higher chitosan content might be attributed to the tendency of the polymer matrix to create a more compact assembly of the particulate components [44–45]. The high porosity is also reflected in the nitrogen adsorption/desorption isotherms (Figure S2a,b in Supporting Information File 1). The BET specific surface area of the samples Film-1 and Foam-1 was 5 and 58 m^2^·g^−1^, respectively.

**Table 1:** Apparent, skeletal, and relative density together with the corresponding porosity of the bionanocomposite foams.

sample	NC^a^ (wt %)	GNP^b^ (wt %)	CHI (wt %)	ρ_app_^c^ (g·mL^−1^)	ρ_sc_^d^ (g·mL^−1^)	ρ_rel_^e^	porosity (%)

Foam-1	18	55	15	0.071	1.9	0.04	96
Foam-2	12	36	45	0.072	1.4	0.05	95
Foam-3	10	30	54	0.076	1.2	0.06	94
Foam-4	6	18	72	0.080	0.7	0.1	89
Foam-5	2	6	91	0.081	0.4	0.21	79

^a^NC = total amount of nanoclays; the ratio between both clay minerals (SEP/HNTs) was kept at 1:1. ^b^The ratio between both nanocarbons (GNPs/MWCNTs) was kept at 5:1. ^c^ρ_app_ denotes the apparent density. ^d^ρ_sc_ denotes the skeletal density. ^e^ρ_rel_ denotes the relative density calculated as ρ_app_/ρ_sc_.

The microstructure of the films was characterized by X-ray diffraction (XRD). The diffractogram of Film-1 displays the main reflections of both nanoclays and GNPs without 2θ displacement (Figure S3, Supporting Information File 1). This suggests that, in contrast to other polymer–HNT composites, no intercalation of chitosan into the halloysite interlayer spacing occurred, and thus, halloysite still remains in its dehydrated form (Figure S4, Supporting Information File 1) [30,34]. Furthermore, a change in the relative intensity of the main halloysite reflections is observed as a typical consequence of a preferential in-plane orientation of the nanotubes in the film architecture (Figure S4, Supporting Information File 1) [46].

**Figure 4:**
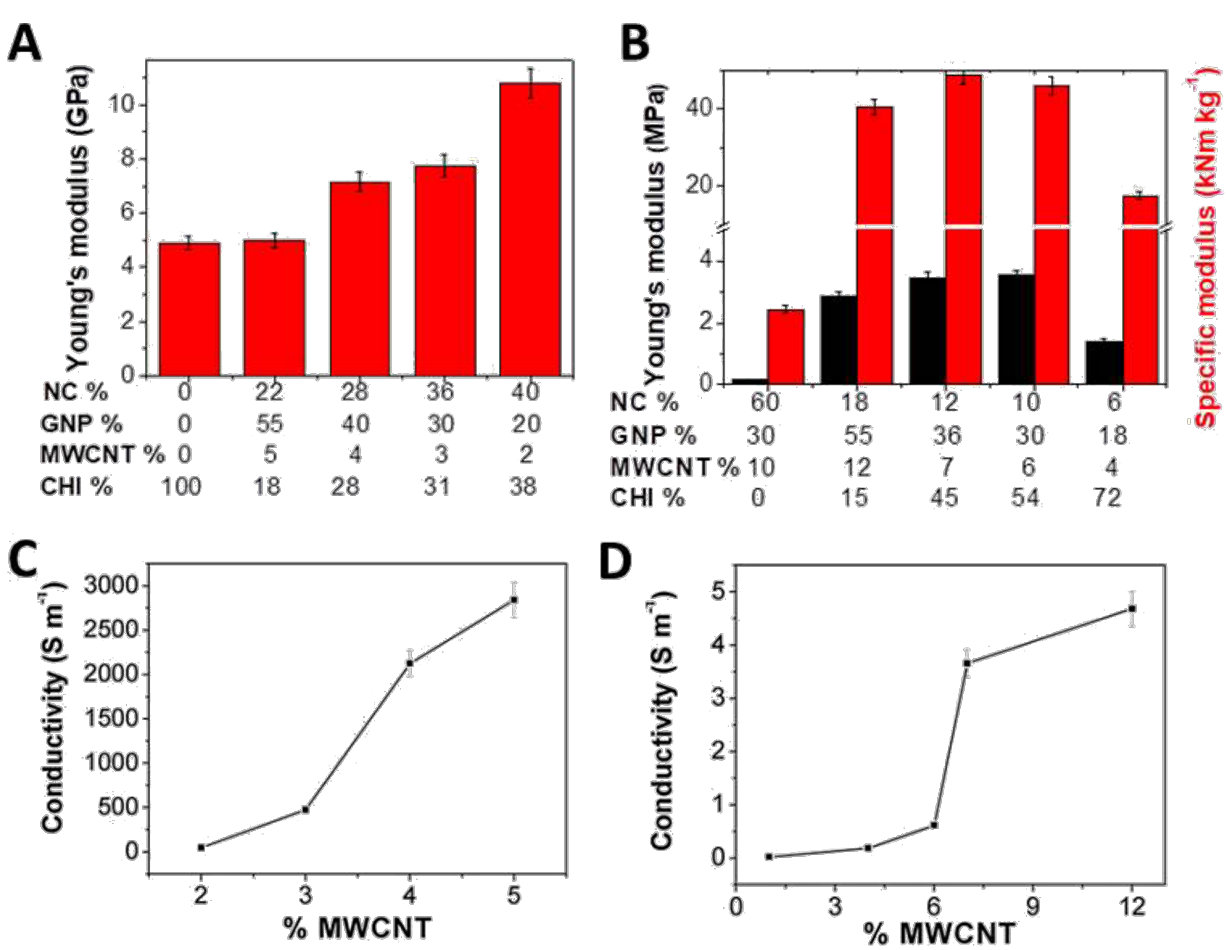
Young’s moduli and electrical conductivity of HNTs/SEP/GNPs/MWCNTs/CHI films (A, C) and foams (B, D), respectively.

The mechanical properties of the bionanocomposite materials were evaluated in stress–strain measurements (Figure S5, Supporting Information File 1), analysing the influence of the nanofiller content on the elastic behaviour as it has been described in related biopolymer-based nanocomposites [26]. The results show that the Young’s modulus of the films (Figure 4A) increases with the clay nanofiller content from 5 GPa for pure chitosan up to 11 GPa for the sample Film-4, which contains 40% of clay components.

These findings are in good agreement with the mechanical properties of similar composite materials based on sepiolite, MWCNTs and poly(vinyl alcohol) [25], sepiolite, graphene nanoplatelets, and biopolymers (e.g., alginate, gelatine) [26] and cellulose or foams of microfibrillated cellulose and starch [47], which exhibit Young’s moduli in the range from 0.1 to 9 GPa. The high stiffness of these materials has been previously attributed to the sepiolite fibres that strongly interact with chitosan chains and may also interlock hindering physical movement and sliding of the other particulate components [48].

The mechanical test (Figure 4B) of the bionanocomposite foams confirmed the crucial role of the chitosan matrix conferring robustness to these systems as the Young’s modulus of 0.2 MPa for a foam without chitosan (with the composition of 1:1:1:0.3 in HNTs/SEP/GNPs/MWCNTs) increases to 3.5 MPa after incorporation of the biopolymer (45 wt %). This increase can be correlated to the strong interaction between the chitosan matrix and the sepiolite fibres as well as to an increase in the relative density that produces a decrease in porosity, commonly related to a smaller pore size and a lower tendency to collapse than in the case of larger macropores [48–49]. In contrast, a decrease in the compression modulus (1.4 MPa) was found for the sample with a higher content of chitosan (72%), suggesting a synergic effect of both clays as reinforcing fillers of the polymer and as adhesive agent, which is required to improve the mechanical properties of the designed samples [50–51]. The obtained compression modulus is comparable to values measured for other chitosan/clay foams (1.4 MPa) [51] and significantly higher than those of self-assembled graphene hydrogels (0.03–0.3 MPa) [52]. Notably, the specific modulus of the bionanocomposite foams was 50 kNm·kg^−1^, which is considerably higher than values reported for silica aerogels (5–20 kNm·kg^−1^) [53] and is on par with polystyrene foams (10–100 kNm·kg^−1^) [49] and other bionanocomposite graphene–clay foams (77 kNm·kg^−1^) [43].

A high electrical conductivity of the bionanocomposite films and foams is crucial for their application in electrochemical devices. The conductivity was therefore assessed by the van der Pauw method based on the four-point technique [26]. This method is useful to accurately measure the surface properties of a sample of arbitrary shape. Figure 4C displays the in-plane electrical conductivity of the films as a function of the MWCNT content (composition of samples in Table 2).

**Table 2:** Composition and nomenclature of the prepared samples.^a^

	sample	NC^c^ (wt %)	GNP^d^ (wt %)	CHI (wt %)

films	Film-1^b^	22	55	18
	Film-2	28	40	28
	Film-3	36	30	31
	Film-4	40	20	38
foams	Foam-1^b^	18	55	15
	Foam-2	12	36	45
	Foam-3	10	30	54
	Foam-4	6	18	72
	Foam-5	2	6	91

^a^The sepiolite (SEP)/halloysite (HNTs) ratio was kept at 1:1. ^b^GOx loaded into HNTs for biosensor and EBC assays. ^c^NC = total amount of nanoclays. The ratio between both clay minerals (SEP/HNT) was kept at 1:1. ^d^In the film composition the ratio between both nanocarbons (GNPs/MWCNTs) was kept at 10:1, whie in the foam it was 5:1.

A remarkable value of 2900 S·m^−1^ is obtained at 5 wt % of carbon nanotubes, while the percolation threshold for electrical conductivity is at 4 wt % MWCNT content. The conductivity values are higher than the values reported previously for sepiolite–nanocarbon–polymer bionanocomposites (1000–2500 S·m^−1^ [14–15]). The high in-plane conductivity found here can be attributed to a synergic effect of MWCNTs and the lamellar assembly of graphene nanoplatelets in the plane of the film as observed by SEM (see Figure 3F). The MWCNTs act as nanowires connecting the GNPs, which facilitates the electron percolation across the insulating network of polymer and clay components [26,29]. In addition, the polymer matrix appears to have a significant influence on the electrical conductivity. While having a similar GNP/MWCNT content bionanocomposites with different polymer matrices showed different conductivity values, i.e., 2700 S·m^−1^ for alginate, 900 S·m^−1^ for gelatin, and 300 S·m^−1^ for poly(vinyl alcohol), and the chitosan matrix discussed here yielded conductivity of 2900 S·m^−1^ [26]. The increase of the conductivity in chitosan films can tentatively be ascribed to the presence of physically adsorbed water not only on the nanoclay surfaces. it might also be associated with the polymer matrix enhancing electrical conductivity trough ionic species and proton diffusion [54–55].

The electrical conductivity of the bionanocomposite foams is presented in Figure 4D. The foams displayed conductivity values of ca. 4.5 S·m^−1^, which is significantly lower than that of films of similar composition. This is attributable to the higher porosity and separation of the charge carriers. However, the electrical conductivity of these foams is considerably higher than that of other related graphene-based foams (0.5 S·m^−1^ [52]). The electrical percolation threshold of the foams was around 6.5 wt % MWCNT content. The higher value in foams reflects a poorer connectivity between carbon nanoparticles dispersed in the clay–polymer matrix probably due to the high porosity, requiring a larger amount of GNPs/MWCNTs to form a conducting network within the matrix of the bionanocomposite. In any case, the percolation threshold is on par or slightly lower than the values for related MWCNT–polymer composites, which are in the range of 4–9 wt % [56–57].

The bionanocomposite sample Film-1 was used to evaluate the stability of these multicomponent hybrid materials in water showing a mass loss of only 3.2 wt % over the course of two months. This excellent stability, together with the good electrical and mechanical properties, suggest that the prepared multicomponent bionanocomposite can be suitable as electrode material in aqueous media. Moreover, the successful incorporation of HNTs as nanoreactor prompted the use of these bionanocomposite materials in bioelectrocatalysis applications (see below).

### Immobilization of glucose oxidase in the lumen of HNTs

The developed multicomponent bionanocomposites were used for the immobilization of the enzyme glucose oxidase in the search of multifunctional properties of interest in bioelectrochemical applications. GOx was chosen as a prototypic bioactive component because of its properties and compatibility with HNTs, i.e., an appropriate size (5.4 nm) as well as an appropriate isoelectric point (at pH 4.0–4.5) for inclusion and immobilization at the surface of the halloysite lumen. Then, HNTs were exploited as nanocontainers for GOx, avoiding the direct interaction the protein with the sepiolite fibres that may lead to enzymatic inactivity [58–59]. In fact, assays showed a drastic loss of enzymatic activity when GOx was incorporated in the film without previous immobilization within HNTs. Hence, GOx was immobilized in the HNT clay prior to its incorporation into the multicomponent mixture. The uptake of GOx was 7.7 ± 0.2 wt % according to CHN elemental analysis. The enzyme immobilization was also confirmed by FTIR spectroscopy (Figure S6 and Table S1, Supporting Information File 1). The HNT–GOx spectrum clearly shows the presence of bands assigned to the symmetric stretching of C–H aliphatic groups and the amide groups of GOx [60]. In particular, there is no significant variation in the amide I and amide II vibrations of the immobilized GOx enzyme with respect to unsupported GOx. This observation strongly supports that the adsorption of GOx in HNTs occurs via non-deteriorating electrostatic interactions [57]. This physical entrapment, in contrast to immobilization via covalent bonding is essential for the preservation of the enzyme structure and bioactivity [38,58].

The presence of GOx in the lumen of HNTs was also evidenced by measuring nitrogen adsorption/desorption isotherms (Figure S7, Supporting Information File 1). Compared to pristine HNTs, a notable decrease of the specific surface area from 25 to 19 m^2^·g^−1^ for HNT–GOx could be observed. The volume of the mesopores was also reduced after GOx uptake (Table S2, Supporting Information File 1) in agreement with a partial pore blockage, supporting the hypothesis that the majority of GOx was loaded into the lumen of HNTs [24].

With the GOx-loaded HNTs a multicomponent bionanocomposite film (Film-GOx) and foam (Foam-GOx) were prepared with the composition of Film-1 and Foam-1, respectively (see Table 2). The enzymatic activity of Film-GOx was confirmed in a test with peroxidase and 2,2'-azino-bis(3-ethylbenzothiazoline-6-sulphonic acid (ABTS), indicating that the preparation procedure did not affect the response of the entrapped GOx towards glucose (Figure S8, Supporting Information File 1).

### Detection of glucose with a film-GOx biosensor

**Figure 5:**
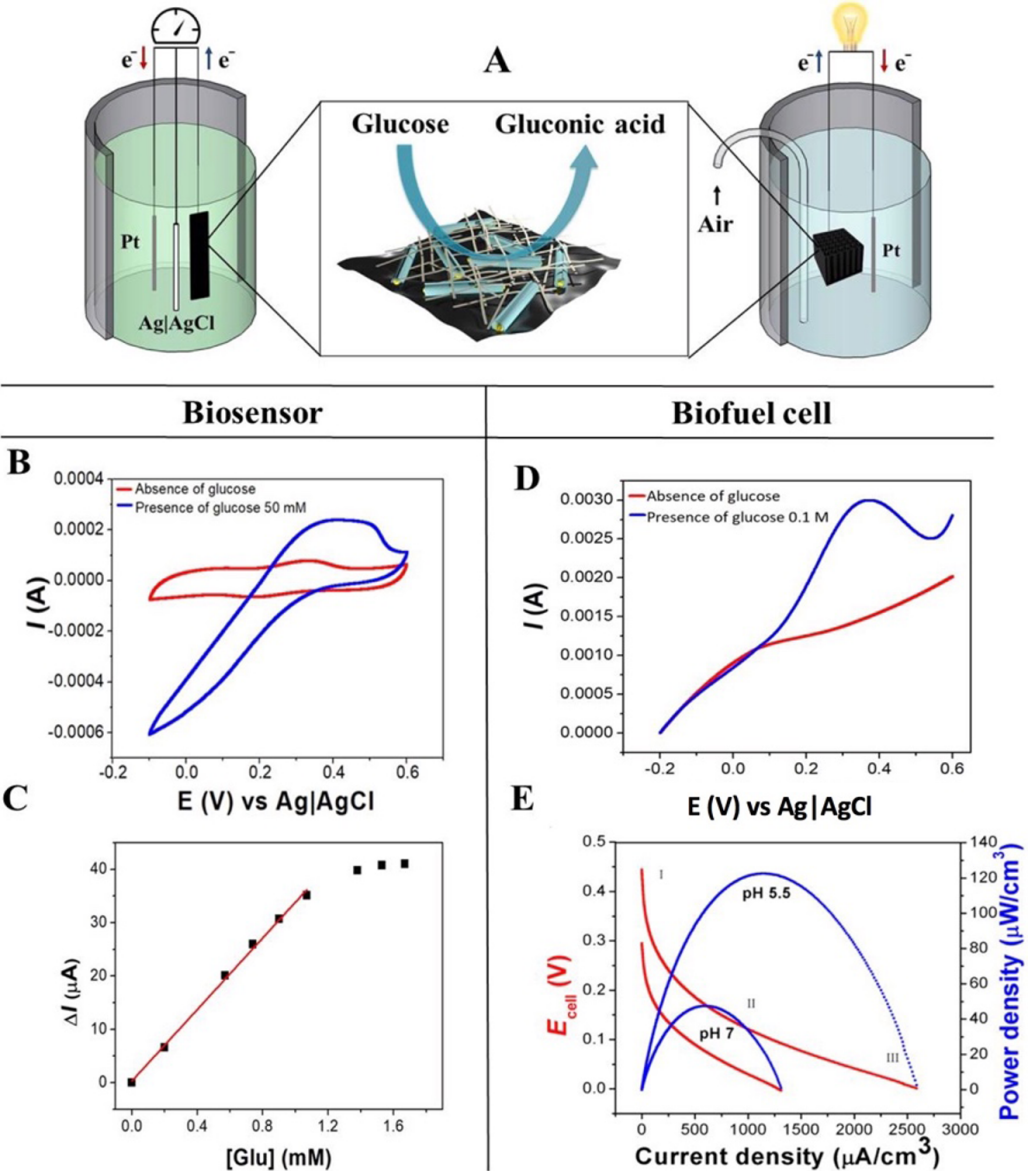
A) Scheme of an EBC (left) and a biosensor (right) with the electrode microstructure and biocatalytic oxidation of glucose at the bioactive nanocomposite interface. B) Effect of glucose on the Film-GOx sample in CV measurement in PBS, pH 7 and 0.1 mM of potassium ferricyanide at a scan rate of 5 mV·s^−1^. C) Sensor response as a function of the glucose concentration. The red line is a linear fit. Δ*I* = steady-state current at 0.45 V. D) LSV measurement of Foam-GOx immersed in PBS at pH 7 and in the presence of 0.1 M glucose in PBS at the same pH vaue. E) Polarization curve obtained by LSV measurements at a scan rate of 1 mV·s^−1^. The medium is PBS with 1 M glucose at pH 7 and pH 5.5.

The GOx-loaded bionanocomposite film (Film-GOx) was tested as biosensor for the detection of glucose (Figure 5A).

The performance of the biosensor was studied by cyclic voltammetry (CV) in the presence of potassium ferricyanide as mediator, relying on the mediated electron transfer (MET) mechanism. Figure 5B shows the CV curve of the biosensor in response to 50 mM glucose in phosphate-buffered solution (PBS). The intensity of the oxidation and reduction peaks of Fe(CN_6_)^4−^ at 0.19 and 0.33 V, respectively, increases significantly in presence of glucose. Together with the change of the CV curve shape this confirms the catalytic behaviour of the immobilized enzyme [61–62]. The steady-state current as a function of the glucose concentration is depicted in Figure 5C showing Michaelis–Menten behaviour, i.e., an effect of the substrate concentration on the rate of the enzyme-catalysed reaction. The use of potassium ferricyanide as mediator enabled a fast electron transfer between the enzyme and the electrode surface. In fact, a fit of the curve with the Lineweaver–Burk plot (Figure S9, Supporting Information File 1) rendered a Michaelis–Menten constant (*K*_m_) of 9.3 mM, which is smaller than those reported for GOx in solution (33 mM) [62] and GOx immobilized in mesopores of Al_2_O_3_ membranes (10–30 mM) [63], sol–gel-derived composite films (14 mM) [64], and similar devices based on graphene and carbon nanotubes (4–15 mM) [65–66]. The low *K*_m_ value is indicative of an excellent performance attributed to strong substrate binding and high enzymatic activity of the immobilized GOx [65].

The linear range of the biosensor was 0–1.1 mM glucose and the sensitivity was as high as 34 µA·mM^−1^. These results also reflect the stronger response of the designed biosensor to low amounts of glucose with respect to other devices based on immobilized GOx on, for instance, graphene, CNTs, and buckypapers (10–25 µA·mM^−1^) [46,66–67], the external surface of functionalised HNTs (5.2 µA·mM^−1^) [20], a polymeric matrix (5 µA·mM^−1^) [68] or a chitosan-modified matrix (1.2 µA·mM^−1^) [69].

The crucial role of HNTs as protective containers for the enzymes was underlined by immobilizing GOx directly on bionanocomposite films prepared without the incorporation into halloysite, where the enzyme was directly integrated in the system after the ultrasonication treatment. The CV curves of these films showed no response to glucose (Figure S10, Supporting Information File 1), suggesting the inactivity of the entrapped enzyme. This is probably due to the direct interaction of the enzyme with sepiolite and shows the necessity to load the enzyme into the clay nanotubes. It is well known that the electrostatic interaction of proteins with the external surface of sepiolite can be very strong and, in some cases, might cause the loss of the biological functionality [60].

### Application of foam-GOx as anode in a membrane-less and open-air biofuel cell

It is well known that redox mediators are required for most of the GOx-based bioelectrocatalysis applications to guarantee an efficient electron transfer process from the enzyme to the electrode interface [70]. Therefore, in a preliminary assay, Foam-GOx was tested in the presence of Fe(CN_6_)^4−^ as mediator and separated from the cathode chamber by a Nafion® membrane. A power density of 565 µW·cm^−3^ and 31 µW·cm^−2^ was generated (Figure S11, Supporting Information File 1). Next, the EBC performance was evaluated under open-air conditions and in the absence of any mediator or expensive proton exchange membranes. Figure 5A illustrates the EBC designed as a one-pot cell. The GOx enzyme catalyses the conversion of glucose in gluconic acid as follows [71]:

[1] $$\text{FAD-GOx + glucose }\overset{}{\to }{\text{FADH}}_{\text{2}}\text{-GOx + gluconic acid}\;\text{.}$$

The reaction occurring on the anode surface is:

[2] $${\text{FADH}}_{2}\text{-GOx }\overset{}{\to }{\text{FAD-GOx + 2H}}^{+}{\text{+2e}}^{-}\;\text{,}$$

while the Pt cathode catalyses the reaction:

[3] $${\text{O}}_{2}+4{\text{H}}^{+}+4{\text{e}}^{-}\;\overset{}{\to }{\text{ 2H}}_{2}\text{O}\;\text{.}$$

Linear sweep voltammetry (LSV) experiments were carried out to evaluate the electrocatalytic properties of the bionanocomposite Foam-GOx as 3D bio-anode. Figure 5D displays the catalytic behaviour during the oxidation of glucose. In fact, with the addition of 0.1 M glucose, a clear increase (blue line) of the anodic current appears compared to that in PBS without glucose. For the here designed mediator-less cell, this behaviour is correlated to a direct electron transfer mechanism at the interface between the active site (FAD) of the enzyme and the conducting elements of the electrode surface [72–73]. The current increase occurs at a voltage of 0.35 V, which is higher than the typical FAD/FADH_2_ standard voltage (−0.460 V at pH 7.0 and 25.8 °C) probably because of the presence of carbon nanotubes that can influence the electrochemical response [60].

The polarization measurements were carried out in a concentrated glucose solution (1 M) to estimate the maximum power density regardless of the glucose content [70–71]. The polarization curves for the described biofuel cell working at two different pH values are shown in Figure 5E. The polarization curves show the common behaviour of microbial fuel cells (MFCs) and EBCs and can be divided into three zones as shown in Figure 5E, commonly called the activation zone (I), ohmic losses (II), and the mass-transport zone (III) [72–73].

The open-circuit potential (OCP) of the cell at pH 5.5 was 0.442 V, while at pH 7 the OCP was 0.298 V. This finding can be correlated to the combination of effects such as a more suitable working pH value for glucose oxidase (the optimal working pH value of GOx is close to 5) and a faster oxygen reduction at the cathode surface. The presence of the acidic medium, in fact, can favour the proton migration from the anode to the cathode surface leading to an increase of the half-cell potential [70]. The power output of the EBC was different for the two pH values. Compared to pH 7 the cell working at pH 5.5 exhibits an increase in volumetric power density from 47.8 µW·cm^−3^ at 0.081 V to 120 µW·cm^−3^ at 0.116 V and in current density from 1.3 mA·cm^−3^ to 2.6 mA·cm^−3^, respectively, as well as a rise in surface power density from 2.6 µW·cm^−2^ to 6.5 µW·cm^−2^. These values are in good agreement with other EBC systems, indicating a good performance the Foam-GOx bionanocomposite (Table 3).

**Table 3:** Performance of different published EBFC systems utilizing DET or MET mechanisms.

anode	cathode	mechanism	OCP (V)	power density	reference

GOx-graphene/SWCNT co-gel	BOD-graphene/SWCNT co-gel	DET	0.61	190 µW·cm^−2^ 650 µW·cm^−3^	[72]
graphite/GOx/catalase/ubiquinone	graphite/PPO/quinhydrone	MET	0.27	24 µW·cm^−3^	[74]
CNT/GOx/catalase	CNT/laccase	DET	0.57	193 µW·cm^−2^ 161 µW·cm^−3^	[75]
Fc-MeOH/GOx CNPs	ABTS2-/BOD CNPs	MET	0.50	95 µW·cm^−2^	[76]
GOx/SWNT/Ppy composite	tyrosinase/CNPs/Ppy composite	DET	—	158 µW·cm^−3^	[77]
CDH/AuNPs	MvBOx/AuNPs	DET	0.57	1 µW·cm^−2^	[78]
GMC/GOx/GA	Pt	DET	0.48	22 µW·cm^−2^	[79]
CNTs/FcMe2-LPEI/Lactate	CNTs/Ar-pyr/BOx	MET	0.44	2.4 µW·cm^−2^	[80]
Foam-GOx	Pt	MET	0.32	31 µW·cm^−2^ 565 µW·cm^−3^	this work
Foam-GOx	Pt	DET	0.44	6.5 µW·cm^−2^ 120 µW·cm^−3^	this work

The decrease of power density compared to the cell working in presence of redox mediator is associated with a slower electron transfer at the enzyme–electrode interface. Nevertheless, the confined GOx in halloysite nanotubes was able to operate even in the DET mechanism, allowing for a use in physiological environments [72,74].

Furthermore, the high surface area promotes a better contact between glucose and the active sites of the enzyme, but at the same time the high porosity of 96% of the bio-anode helps to delay the leaching of bioactive components. Before being released into the surrounding medium, the enzyme must take a tortuous path through the pore system of the foam, resulting in a good stability over time. A preliminary evaluation of this effect was carried out by repeating the test after a period of five days of storage in PBS at 30 °C, after which 93% of the initial power was retained.

## Conclusion

This work reports a preliminary study showing the viability to integrate nanoclays, biopolymers, and graphene-based conducting components into homogeneous multifunctional nanoarchitectured materials. The presence of sepiolite fibrous clay, together with ultrasonication, is the key to disperse all these components in water. The resulting stable colloids can be processed as films and foams displaying acceptable mechanical properties, good electrical conductivity, and controlled porosity useful for diverse applications at the nanoscale. HNTs were efficiently loaded with 7.7 wt % of the model enzyme glucose oxidase that retained high enzymatic activity inside the halloysite lumens. This allows for the exploration of the multicomponent bionanocomposites as functional components of electrochemical devices such as biosensor and as 3D bio-anode in a biofuel cell. The latter revealed a volumetric power density of 120 µW·cm^−3^ and a good stability over time at elevated temperatures (the power density decreased by only 7% after five days of storage at 30 °C). These bioelectrocatalysis results are representative for an incipient development that could be sextended in the future to other fields of interest, especially considering the versatility of halloysite as nanocontainer of various bioactive species [17]. The possibility to introduce additional functionalities by modification of sepiolite, for instance, by incorporating magnetic or photoactive nanoparticles [8], could pave the way to further applications of these multicomponent functional bionanocomposites in the near future.

## Experimental

### Materials

Sepiolite (SEP) from the Vallecas-Vicálvaro clay deposits (Madrid, Spain) was provided by TOLSA S.A. (Spain) as a commercial, rheological grade product (Pangel^®^ S9). This microfibrous clay has a low cationic exchange capacity (ca. 15 meq·(100 g)^−1^) and high specific surface area (ca. 300 m^2^·g^−1^). Dehydrated halloysite nanotubes (HNTs) from the New Zealand China Clays deposits were provided by Imerys (France). Before use, HNTs were ground and sieved through a 250 µm mesh. Glucose oxidase (GOx; type VII-S, 181,500 U·g^−1^ solid; E.C.1.1.3.4 from Aspergillus niger) was supplied by Sigma-Aldrich. Graphene nanoplatelets (GNPs) are multilayered graphene sheets that were supplied by KNANO (China) under the name of KNG-150. They are composed by more than ten carbon layers with 5–15 nm thickness and 1–20 µm diameter, showing an electrical conductivity of 12000 S·m^−1^ and a specific surface area of 41 m^2^·g^−1^ (according to the manufacturer). Multiwalled carbon nanotubes (MWCNTs), with more than 95% of carbon content, were obtained from Dropsens S.A. (Spain) and used without further treatment. The average diameter of the tubes was 10 nm and the average length 1.5 µm. Acetic acid (ca. 99.5%) was obtained from Merck. Anhydrous D-glucose (99 %) was obtained from Scharlau. Peroxidase (HRP; type II, 120,000 U·g^−1^ solid; E.C.1.11.1.7 from horseradish) were purchased from Sigma Chemical Co., 2,2'-azino-bis(3-ethylbenzothiazoline-6-sulphonic acid) (ABTS) was obtained from Fluka. Trisodium phosphate dodecahydrate (ca. 98%), was furnished by Sigma and phosphoric acid (85%) by Carlo Erba. Bi-distilled water (18.2 MΩ·cm) was obtained from a Maxima Ultra Pure Water system from Elga. Chitosan with a medium molecular weight of 190–310 kDa, 75–85% deacetylated, was obtained from Aldrich.

### Preparation of colloidal suspensions and films and foams

The preparation of the multicomponent bionanocomposites is shown in Figure 1. Two sets of aqueous mixtures of chitosan (CHI) and different proportions (Table 2) of SEP/HNTs/GNPs/MWCNTs were prepared at overall concentrations of 0.2% w/v and 8% w/v, respectively. First, the appropriate amounts of both nanoclays and GNPs/MWCNTs were dispersed in bi-distilled water and exposed to pulsed ultrasonic waves (VC750 Sonics Vibra-Cell, operating at 20 kHz) using a 13 mm standard probe. Separately, chitosan was slowly dissolved in an aqueous solution of 1% v/v acetic acid at 70 °C and added to the SEP/HNTs/GNPs/MWCNTs dispersion under magnetic stirring.

The bionanocomposite films were processed by solvent-casting from the 0.2% w/v dispersion on polyester Petri dishes and dried at 30 °C and 60% relative humidity (RH) in a CLIMACELL EVO Stability Chamber (Incubator model 111L).

The bionanocomposite foams were prepared by freeze-drying (Cryodos-80, Telstar) of the 8% w/v dispersion, which was cast in cylindrical plastic containers and plunged in liquid nitrogen.

### Immobilization of glucose oxidase in halloysite nanotubes and their incorporation in bionanocomposite matrices

Glucose oxidase (100 mg) was dissolved in water (1 mL) and mixed with HNTs (200 mg). Then, the sample was vortexed and sonicated in an ultrasound bath until no aggregates of halloysite were visible. In order to ensure the complete infiltration of the HNT lumens by the GOx solution the samples were subject to alternating cycles of reduced pressure (approx. 70 Torr). The loaded HNT-GOx was separated from the solution by centrifugation, washing and finally dried overnight in a desiccator at 30 °C and stored at 4 °C until usage. HNT-GOx was added to the SEP/GNPs/MWCNTs/CHI mixtures (0.2% w/v and 8% w/v) described above, obtaining the compositions Film-1 and Foam-1 (Table 2). The resulting suspensions were processed by solvent-casting and freeze-casting to obtain the bioactive films (film-GOx) and the bioactive foams (foam-GOx), respectively, and were stored at 4 °C until usage.

### Characterization techniques

The morphology of the prepared bionanocomposite films and foams was evaluated by scanning electron microscopy (SEM) using a SEM Philips XL 30 S-FEG microscope. Before examination, the samples were fractured in liquid nitrogen. The FTIR spectra of HNT-GOx samples were acquired with a BRUKER iFS spectrophotometer 66Vs. X-ray diffractograms were obtained with a D8-ADVANCE diffractometer (Bruker), using Cu Kα radiation. The voltage and current sources were set at 40 kV and 30 mA, respectively. Diffractograms were recorded at a goniometer speed of 0.5 s per step between 4° and 60° (2θ). The BET specific surface area and the pore size distribution (Barret–Joyner–Hallenda method) were determined from nitrogen adsorption/desorption isotherms obtained on a Micromeritics ASAP 2010 analyser. The samples were degassed at 120 °C under vacuum. The stability of the bionanocomposites in water was assessed by immersing a piece of the film in bi-distilled water for two months and noting the weight loss. The relative density (ρ_rel_) of the bionanocomposite foams was estimated from the skeletal density using the following values: SEP = 2.3 g·mL^−1^, HNTs = 2.2 g·mL^−1^, GNPs = 2.3 g·mL^−1^, MWCNTs = 2.1 g·mL^−1^, chitosan = 0.2 g·mL^−1^. The mechanical properties of the films and foams were assessed under ambient conditions by using a universal test machine (Instron Model 3345) equipped with a 5 kN load cell and at 1 mm·min^−1^ frame speed. At least three measurements were performed per sample. The electrical conductivity was determined by the four-point method, using a Solartron 1480 potentiostat (MultiStat). Elemental chemical analysis (CNHS Perkin Elmer 2400 analyzer) was carried out to estimate the amount of GOx loaded into the HNTs.

### Biosensing test

Cyclic voltammetry (CV) was performed with a standard three-electrode electrochemical cell connected to a Solartron 1480 MultiStat potentiostat. A platinum wire was used as a counter electrode and Ag|AgCl (soaked in 1.0 M KCl) was used as a reference electrode. In the biosensing tests, the working electrode was a film of 30 × 5 mm × 0.014 (3.49 mg, containing 0.028 mg of immobilized GOx) immersed in a potassium ferricyanide solution (0.2 mM) as mediator containing 0.1 M of phosphate buffered solution (pH 7). CV was performed in a potential range from −0.2 to 0.6 V at a scan rate of 5 mV·s^−1^.

### Biofuel cell test

Polarization curves were obtained from linear sweep voltammetry performed with a μStat 100 potentiostat (Dropsens, Spain) in a two-electrode configuration. The glucose/air biofuel cell was assembled by coupling the bioactive foam, as anode, to a Pt wire as cathode in a one-pot cell working in 0.1 M of glucose and 0.1 M PBS, at two different pH values (7 and 5.5) and saturated with air. The foam was connected to the potentiostat with a copper wire, glued with colloidal graphite and covered by an epoxy resin as isolating material. All tests were run three times at a scan rate of 1 mV·s^−1^ starting from the open-circuit potential (OCP, *I* = 0) to the short-circuit cell voltage (*I* = *I*_max_). From the data of V_cell_ as a function of *I*, the power (P) was calculated according to Equation 4.

[4] $$P=I\cdot V_{\text{cell}}\;.$$

Finally, the power density was obtained as a surface power density (μW·cm^−2^) with the roughness factor (ECSA) calculated from the CV measurements, and as a volumetric power density (μW·mL^−1^) considering a specific volume (0.02 cm^3^), calculated from the specific density (1.9 g·mL^−1^ ) [29].

## Supporting Information

File 1 Additional experimental data.
