# Supplementary material for: Multicomponent bionanocomposites based on clay nanoarchitectures for electrochemical devices
Source: Beilstein J Nanotechnol. 2019 Jun 25;10:1303–15. doi: 10.3762/bjnano.10.129 (PMC6604714; doi:10.3762/bjnano.10.129)
Supplement: File 1 — Additional experimental data. [file Beilstein_J_Nanotechnol-10-1303-s001.pdf]

BEILSTEIN JOURNAL OF NANOTECHNOLOGY

Supporting Information

for

Multicomponent bionanocomposites based on clay nanoarchitectures for electrochemical devices

Giulia Lo Dico, Bernd Wicklein, Lorenzo Lisuzzo, Giuseppe Lazzara, Pilar Aranda and Eduardo Ruiz-Hitzky

Beilstein J. Nanotechnol. **2019,** *10,* 1303–1315. doi:10.3762/bjnano.10.129

Additional experimental data

License and Terms: This is a supporting information file under the terms of the Creative Commons Attribution License (http://creativecommons.org/licenses/by/4.0). Please note that the reuse, redistribution and reproduction in particular requires that the authors and source are credited.
The license is subject to the *Beilstein Journal of Nanotechnology* terms and conditions: (https://www.beilstein-journals.org/bjnano)

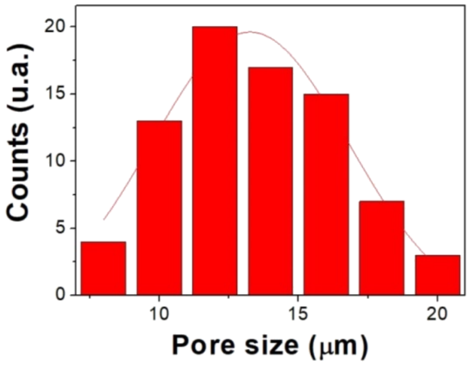

Figure S1: Pore size distribution of the bionanocomposite Foam-1 obtained by image analysis of SEM cross-section micrographs.

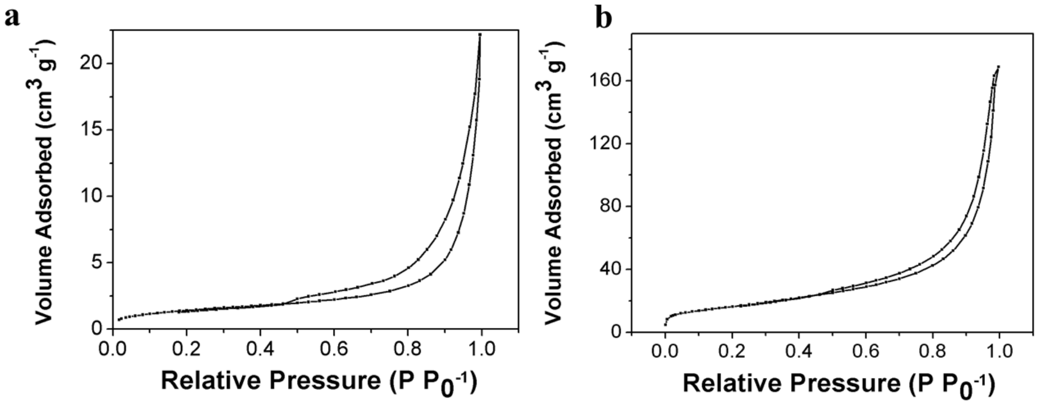

Figure S2: N_2 adsorption/desorption isotherm of bionanocomposites: a) Film-1 and b) Foam-1.

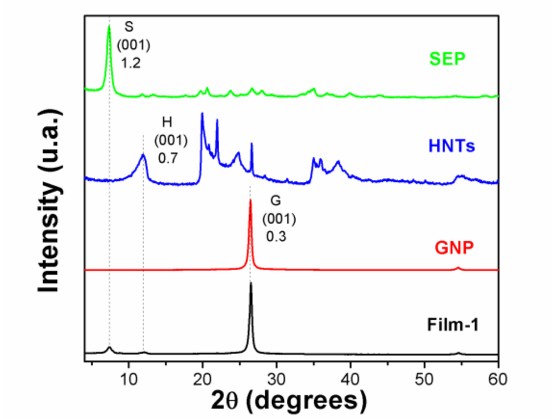

Figure S3: XRD pattern of sepiolite (SEP), halloysite nanotubes (HNTs), graphene nanoplatelets (GNPs) and the bionanocomposite film (Film-1). The main reflection planes of sepiolite (S), halloysite (H), and graphene nanoplatelets (G) are shown with the corresponding Miller indices and interlayer distance values (nm).

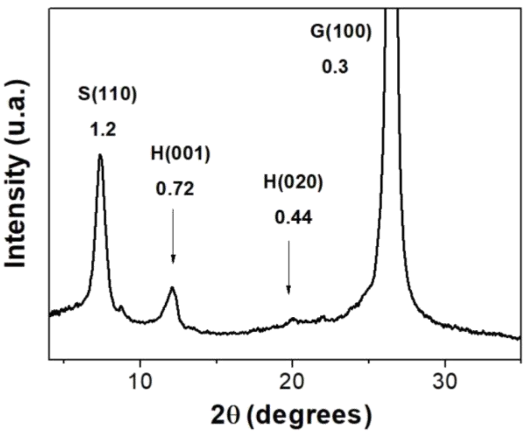

Figure S4: Magnification of the XRD pattern of the bionanocomposite Film-1. The principal reflection planes of sepiolite (S) halloysite (H) and graphene nanoplatelets (G) are shown with the corresponding Miller indices and the interlayer distance values (nm).

Generally, the (001) diffractions are dominant when nanotubes are oriented lying in the plane of the film, while the other diffractions (e.g., the (020) plane) appear as weak reflections. This alignment of the tubes, with their long axis in the plane, increases the intensity of the (001) reflection with respect to the (020) reflection, i.e., the (001)/(020) intensity ratio increases from 0.5 in powder halloysite to 1.8 in the Film-1 [1].

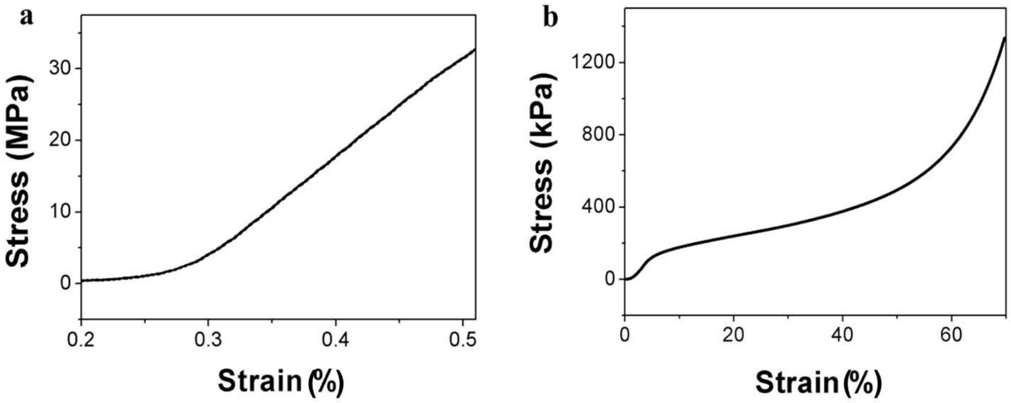

Figure S5: Stress–strain curve of bionanocomposites: a) Film-1 and b) Foam-1.

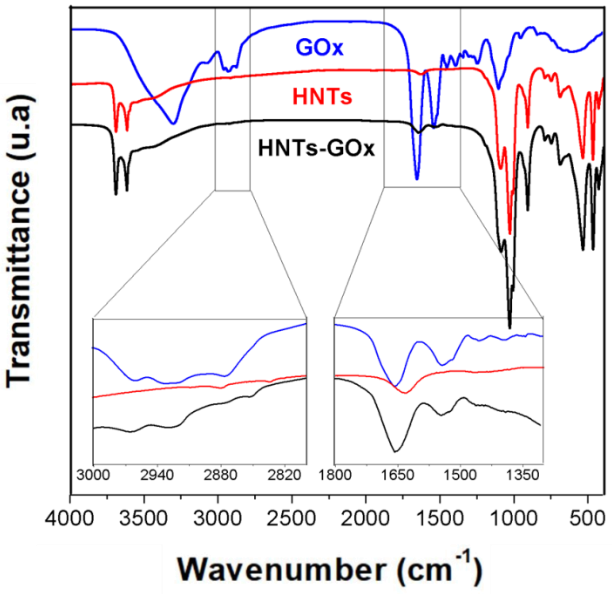

Figure S6: FTIR spectra of pristine GOx, HNT and HNT-GOx, with magnifications in the aliphatic region (3000–2800 cm^{-1}) and double-bond region (1800–1300 cm^{-1}).

Table S1: Summary of the assigned FTIR main bands of the spectrum of HNT–GOx sample.

**position (cm^{-1})**	**assignment**
3695, 3656	O–H stretching of inner-surface hydroxyl group[a]
3620	O–H stretching of inner hydroxyl group
3600, 3450	O–H stretching of water
ca. 2900	symmetric stretching of $C–H_2$
1655	amide I (C=O)
1633	O–H deformation of water in HNTs
1543	amide II (N–H)

[a]The transition moment is nearly perpendicular to the (001) plane.

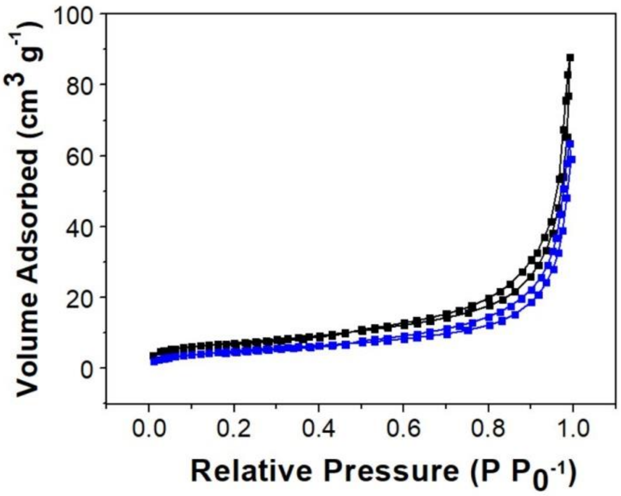

Figure S7: N_2 adsorption/desorption isotherms of pristine HNTs (black curves) and HNTs-GOx (blue curves).

Table S2: Specific BET surface area and the mesopore characteristics of HNTs and HNT-GOx.

	S_{BET}[a] $(m^2 \cdot g^{-1})$	d_{mes}[b] (nm)	V_{mes}[c] $(cm^3 \cdot g^{-1})$	S_{mes}[d] $(m^2 \cdot g^{-1})$
HNT	25.1	11.2	0.063	22.2
HNT-GOx	18.8	11.2	0.046	15.9

[a]Specific surface area calculated by means of the Brunauer–Emmet–Teller (BET) equation in the linear interval of relative pressure from $P \cdot P_0^{-1}$ 0.05 to 0.3.

[b]Average mesopores diameter calculated by using the Barret–Joyner–Hallenda (BJH) method from the isotherm data in the adsorption part.

[c]Average mesopores volume calculated by using the BJH method from the isotherm data in the adsorption part.

[d]Average mesopores surface area calculated by using the BJH method from the isotherm data in the adsorption part.

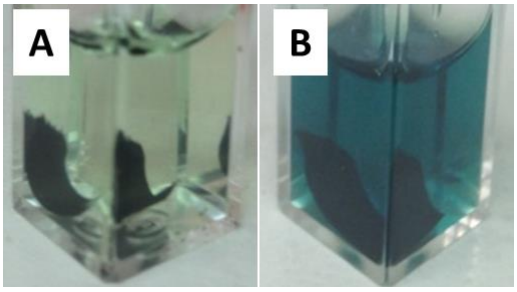

Figure S8: Peroxidase assay of Film-GOx in 800 μL of buffered solution (PBS), 50 μL of ABTS/peroxidase stock (50 mM and 25 units/ml respectively), 100 μL of glucose stock (0.1 M), at a) time $t = 0$, and b) after 10 min.

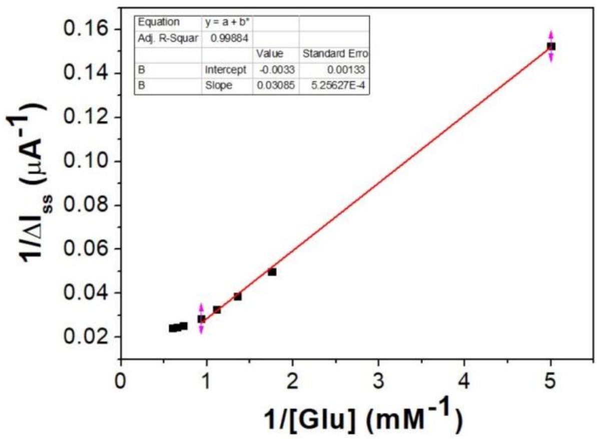

Figure S9: Lineweaver–Burk plot obtained by CV of Film-GOx working in PBS at pH 7 and 0.1 mM of ferricyanide, at a scan rate of 5 mV·s^{-1}.

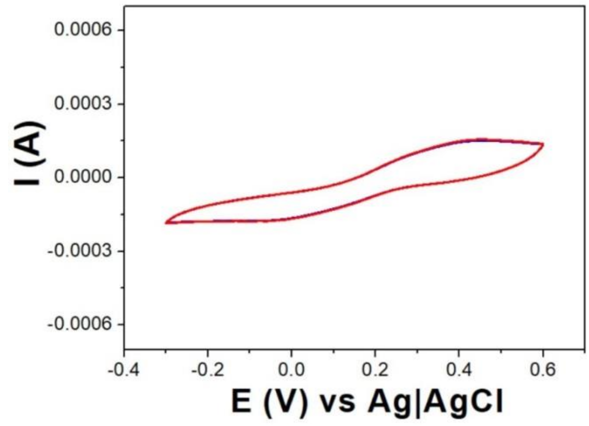

Figure S10: CV of the bionanocomposite film prepared in absence of halloysite nanocontainers. The measurement was acquired in PBS at pH 7 and 0.1 mM potassium ferricyanide and at scan rate of 5 mV·s^{-1}. The red curve is in absence of glucose and the blue one is in presence of 5 mM glucose.

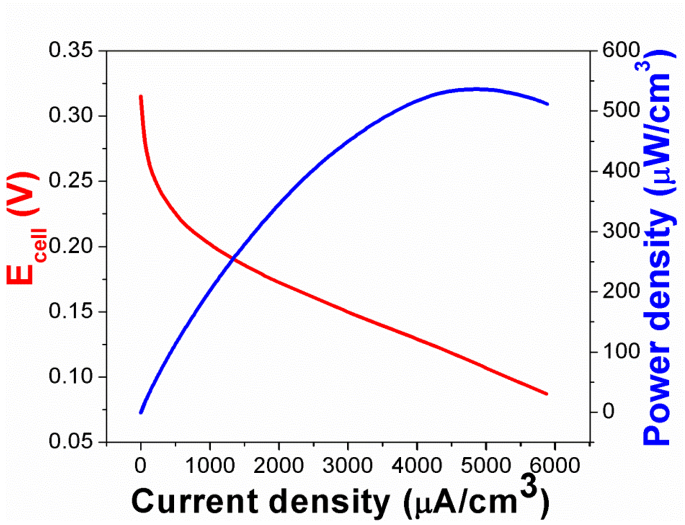

Figure S11: Polarization curve obtained by LSV measurement at a scan rate of 1 $mV{\cdot}s^{-1}$. Foam-GOx was tested in PBS with 1 M glucose and in presence of 0.1 M $Fe(CN_6)^{4-}$. The anode and the cathode chambers were separated by a Nafion® membrane.

References

1. Żbik, M. S.; Raftery, N. A.; Smart, R. S. C.; Frost, R. L. *Appl. Clay Sci.* **2010,** *50*, 299. doi:10.1016/j.clay.2010.08.010
